# Volatile aromatic substances analysis of different temperature stored ‘Docteur Jules Guyot’ pear (*Pyrus communis* L.)

**DOI:** 10.3389/fpls.2025.1572093

**Published:** 2025-04-10

**Authors:** Xiaomeng Shi, Xinxin Zhu, Xiaofei Xu, Xin Zhang, Fudong Jiang, Qingyu Li, Aidi Zhang, Jianzhao Li

**Affiliations:** ^1^ School of Horticulture, Ludong University, Yantai, China; ^2^ Yantai Academy of Agricultural Sciences, Yantai, China; ^3^ School of Food Engineering, Ludong University, Yantai, China

**Keywords:** pear, volatile substance, aroma, temperature, post-harvest

## Abstract

During the post-ripening process of ‘Docteur Jules Guyot’ pear, the aroma of the fruit gradually becomes richer with increasing maturity. In this study, the volatile substances in ‘Docteur Jules Guyot’ pear fruits stored at room temperature (RT), low temperature (LT), and low temperature to room temperature (LT-RT) were identified and analyzed using solid-phase microextraction (SPME) with gas chromatography–mass spectrometry (GC–MS) at different ripening stages. The results showed that the volatile substances were mainly alcohols, aldehydes, esters, acids, ketones, alkanes, and terpenes. Esters are the main substances of fruit aroma; with an increase in fruit maturity, the ester content increases gradually. Ethyl acetate, hexyl acetate, heptyl acetate, and amyl acetate were the main volatile components of the fruit. The aroma content under LT was lower than that under RT, and after transferring from LT-RT, the ripening of the fruit was accelerated, and the aroma content increased rapidly. Among the genes involved in the lox pathway, the expression of *PcHPL1*, *PcADH1*, *PcGLIP1*, *PcGLIP-like*, *PcLOX2*, *PcLIP2*, and *PcFAD2* were the most contributing to the changes of esters in ‘Docteur Jules Guyot’ pear. These results are helpful to provide basic data for the study of volatile in ‘Docteur Jules Guyot’ pear fruit under LT and RT storage.

## 1 Introduction

The ‘Docteur Jules Guyot’ pear (*Pyrus communis* L.) is one of the primary European pear varieties. Currently, it is mainly cultivated in the Liaodong Peninsula and the Yantai area of China. This kind of fruit undergoes ripening and softening when stored at room temperature after harvest and is extremely prone to spoilage. In order to extend the shelf life of European pears and maintain their quality, refrigeration has become the most common and effective storage method. Merchants usually choose to transport and store European pear at low temperatures. When European pears reach the sales location, they are sold at room temperature. During the post-ripening process of ‘Docteur Jules Guyot’ pear, the aroma of the fruit will become gradually rich with the increase of maturity. Aromas are composed various complex volatile aromatic compounds. While consumers choose fruit, in addition to looking at the peel color of the fruit, aroma is also an important factor affecting their choice (Villatoro et al., 2007).

The aroma is an important feature for measuring fruit ripeness and has also been well studied in various fruits. During ripening, the characteristic aroma of fruits such as apples, bananas, and other climacteric fruits is gradually produced, with the peak of volatile compounds occurring during the climacteric spike (Dixon and Hewett, 2000; Watharkar et al., 2020). During ripening, esters, terpenes, aldehydes, and alcohols were the main volatile compounds identified in feijoa fruit. Before fruit ripening, they have a rich grassy aroma, which is mainly attributed to alcohols and aldehydes. As the fruit matures, the ester and terpene content gradually increases, and the fruit aroma becomes increasingly intense (Song et al., 2023). Similarly, volatile compounds in tomatoes also accumulate at the beginning of fruit ripening, with the highest concentration of esters occurring at full ripeness or in the period just before (Karlova et al., 2014). Research has indicated that hexyl acetate, 2-methylpropyl acetate, butyl acetate, butyl butyrate, and pentyl acetate enhance the aroma of pears (Lu et al., 2012). In ‘Nanguo’ pear, esters are the most abundant aroma components (Zhou et al., 2015). After storage for a period of time, ‘Nanguo’ pear fruit reached the best taste period, and the content of ester substances peaked, among which the highest content ester substances were butyl acetate, ethyl acetate, and hexyl acetate (Luo et al., 2021). Similarly, esters are also crucial in strawberries, accounting for up to 90% of the total volatile content, and compounds such as hexanal, trans-2-hexenal, and cis-3-hexen-1-ol are the major components imparting a grassy taste to the fruit (Liu et al., 2023). However, the levels of esters and aldehydes are not only influenced by ripeness but are also closely related to the variety of strawberry (Jetti et al., 2007).

In addition to the genetic composition of the fruit, there are many other factors that affect aroma composition, including ripening conditions, storage environment, and post-harvest treatment (El Hadi et al., 2013; Lee et al., 2018; Zhao et al., 2019). Most volatile organic compounds are produced by metabolic processes during fruit ripening, such as fatty acid, carotenoid, and amino acid metabolism (Tobaruela et al., 2021). The analysis of volatile substances in various fruits revealed that esters are the primary contributors to fruit aroma. Among the biosynthetic pathways of fruit volatile esters, the lipoxygenase (LOX) pathway is the most important for the physiological metabolism of fruits (Wang et al., 2018). Linoleic acid is converted to hydroperoxides by oxygenation, transformed into aldehydes via the lyase (HPL) pathway (Contreras and Beaudry, 2013), reduced to alcohols by alcohol dehydrogenase (ADH), and finally converted to esters by the alcohol acyltransferase (AAT) pathway (Xi et al., 2012). By studying the post-harvest quality of strawberries stored at room temperature (5°C and 10°C) and low temperature (0°C), it was observed that the production of aroma compounds was influenced by storage time and temperature, and the levels of strawberry volatile substances stored at room temperature were higher than those stored at low temperatures (Ayala-Zavala et al., 2004). Melting flesh peaches were stored at 0°C, 5°C, and 8°C for 21 days, revealing that the production of volatile substances was inhibited by low temperatures, and the relative expression levels of genes involved in the LOX pathway were also inhibited (Zhang et al., 2011).

In previous research, it was found that low-temperature storage is one of the most commonly used methods to extend the shelf life of European pear (*P. communis* L.) (Li et al., 2023). Although European pears can be stored for a longer period at low temperatures, they tend to soften rapidly when transferred to room temperature (Dai et al., 2023). Different storage temperatures and rapid temperature changes not only affect the texture of the fruit but are also likely to affect the aroma of the fruit, thereby affecting consumer preference. However, in the case of the ‘Docteur Jules Guyot’ pear, research on the influence of temperature changes on aroma is still limited. Therefore, we analyzed the volatile organic compounds of ‘Docteur Jules Guyot’ pears stored at room temperature (RT), low temperature (LT), and low to room temperature (LT-RT) on different storage days by GC–MS and classified these volatile organic compounds. The results of this study will help provide valuable information on the characteristic aromas of ‘Docteur Jules Guyot’ pear fruit.

## 2 Materials and methods

### 2.1 Plant materials

‘Docteur Jules Guyot’ pear was picked and transported to the laboratory from Yantai Academy of Agricultural Sciences, Yantai City, Shandong Province, on 21 July 2022, and fruit of uniform size, with no mechanical damage to the peel, no diseases and pests, and in good condition were selected as experimental materials.

### 2.2 Treatment

The selected fruit were stored at room temperature (RT, 23 ± 2°C) and low temperature (LT, 2 ± 2°C) respectively, after 10 days of storage at low temperature, the fruit was transferred to room temperature (LT-RT) for further storage. During storage, ensure that the light GRAPH (12-hour light cycle) and humidity (80%) in the storage room are consistent. Nine pears were removed to determine the physiological index. The fruit was cut into pieces, poured into liquid nitrogen, and frozen in an ultra-low-temperature freezer for preservation.

### 2.3 Firmness determination

Fruit firmness was measured using a fruit durometer, the unit was Newton (N), the middle parts of the fruit were selected, the peel of the fruit was cut to a thickness of approximately 1 cm, and the probe of the durometer was pressed into the flesh after being flattened, the line on the probe prevailed, and then the probe was lifted to read the number on the durometer.

### 2.4 Determination of ethylene content

Nine randomly selected pear fruits were placed in a sealed container for 1 h, and then the gas was aspirated from the top of the containers using a 10 mL syringe and repeated three times, which was preserved in a syringe bottle by the drainage method. To determine the ethylene content, a Shimadzu GC-2014 gas chromatography (GC) system (Shimadzu Co., Ltd., Kyoto, Japan) was used with injector, detector, and oven temperatures of 110°C, 150°C, and 70°C, respectively.

### 2.5 L*, a*, b* analysis

L*, a*, and b* values were detected using a CR-400 Color Chroma Meter (Hangzhou Ke Sheng Instrument Co., Ltd.), and a whiteboard was used to calibrate the color before measurement. Nine pear fruits were measured to obtain the L*, a*, and b* values for each fruit, which were expressed as positive and negative numbers.

### 2.6 Determination of TSS and acid of fruit

Two slices of pulp of a certain thickness were cut in the middle part of the fruit, the ends were squeezed, the pear juice was dropped onto an ATAGO hand-held saccharimeter to measure the soluble solids (TSS), aspirate 200 μL of pear juice, add 5 mL of sterile water, shake well, and drop it onto the saccharimeter again to determine the titratable acid (acid) content.

### 2.7 Determination of volatile aromatic substances

The content of volatile compounds in fruit was determined using solid-phase microextraction-gas chromatography-mass spectrometry (SPME-GC-MS), and the determination method was slightly modified based on previous studies (Zhang et al., 2020). Approximately 2.0 g of fruit sample was weighed and placed in a headspace vial. Then, 3 mL of saturated sodium chloride solution was added. 2-octanol was selected as the internal standard, and 5 μL of 2-octanol solution (0.8 mg/mL) was added simultaneously. The mixed solution was vortexed and balanced for 30 min using a magnetic stirrer at 45°C and 260 rpm. Solid-phase microextraction (SPME) fibers [50/30 µm DVB/CAR/PDMS (USA)] were used to collect volatile compounds. The volatile compounds were analyzed using a gas chromatography–mass spectrometry (GC-MS, 7890A + 5975C, Agilent, USA). The gas chromatograph was equipped with a DB-WAX chromatographic column (30 m, 0.32 mm, 0.25 μm). The temperature program began at 40°C, increased to 100°C at 3°C/min, and then to 245°C at 5°C/min. Helium was used as the carrier gas at a flow rate of 1.0 ml/min. The mass spectrometry source was set to 230°C, the column effluent was ionized with an electron energy of 70 eV, and the transfer interface zone was set to 250°C. Qualitative scanning was performed in the range of 35 m/z–350 m/z. Preliminary identification of volatile compounds was accomplished by comparing the measured electron ionization mass spectra with the standard spectra in the NIST-2017 mass spectral library.

### 2.8 RNA extraction, cDNA synthesis, and qRT-PCR

The total RNA of the samples was extracted using the RNAprep Pure Polysaccharide Polyphenol Plant Total RNA Extraction Kit (Tiangen, Beijing, China), was used according to the manufacturer’s instructions. After determining the concentration of RNA, gel electrophoresis was performed to verify the presence of the correct bands, and the extracted RNA was reverse transcribed according to the instruction manual of HIScript II Q RT SuperMIX for qPCR (+gDNA wiper) (Vazyme, Nanjing, China) to obtain cDNA. A mixed RT-qPCR system was added to a 0.1 mL 96-well PCR plate (CELLPRO, Suzhou, China) and placed into a Bio-Rad CFX Real-time Connection System for gene expression analysis.

### 2.9 Data statistics

Data were analyzed using one-way ANOVA in Microsoft Excel 2019. All figures were generated using TBtools, GraphPad Prism 9, and Origin 2022 software (USA). *P indicates significance of t-test with a p-value of <0.05, and three biological replicates were performed independently for each sample.

## 3 Results

### 3.1 Fruit quality analysis

**Figure 1:**
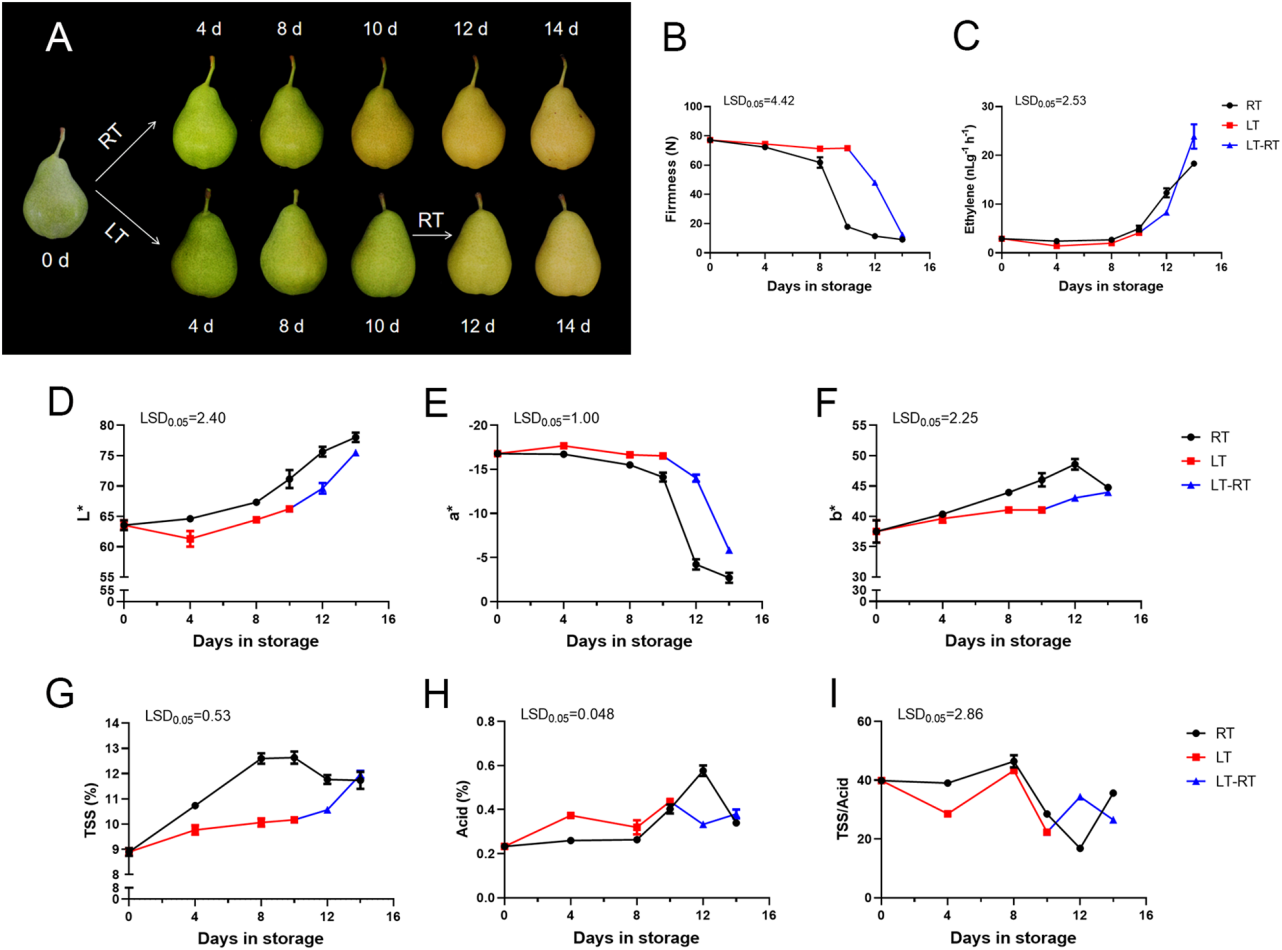
Phenotype and physiological changes of ‘Docteur Jules Guyot’ pear at different temperature. **(A)** The appearance of ‘Docteur Jules Guyot’ pear. **(B)** The firmness of ‘Docteur Jules Guyot’ pear. **(C)** The ethylene content of ‘Docteur Jules Guyot’ pear. **(D–F)** The L*, a*, and b* values of ‘Docteur Jules Guyot’ pear. L*, brightness; a*, reddish green degree (+: red, −: green); b*, yellowish-blue degree (+: yellow, −: blue). **(G–I)** Total soluble solid (TSS), total acid, and TSS/acid contents. Error bars represent three repeated SEs. The observed LSD values suggested the presence of LSD with a significance level of p = 0.05. RT, room temperature (23 ± 2°C); LT, low temperature (2 ± 2°C); and LT-RT, low temperature to room temperature.

The ‘Docteur Jules Guyot’ pear fruit were stored at room temperature (RT), low temperature (LT) and low temperature to room temperature (LT-RT). The fruit stored at RT showed a greenish coloration before 8 days, and a transition from green to yellow was observed between 8 and 10 days of storage. After 14 days, brown spots began to appear on the surface of the fruit, which also indicated that the quality of the fruit gradually deteriorated. In contrast, the fruit stored at low temperature remained green until the 10th day and showed no signs of fading, but after transferring the fruit to RT, it could be seen that the fruit turned yellow rapidly (**Figure 1A**). The firmness of the RT stored fruit decreased rapidly between 8 days and 10 days, from 61.8 N to 17.9 N, which means that the fruits began to ripen rapidly in this period of time. In the case of the fruit at LT, the firmness remained at 71 N. The firmness decreased rapidly, from 71.6 N to 12.4 N, after transfer from LT to RT (**Figure 1B**). The ethylene content of the fruit at RT was significantly higher than that of the fruit stored at LT; however, on the 4 days of the transfer from LT to RT, the ethylene content was significantly higher than that of fruit stored at RT (**Figure 1C**). The values of L* and b* were consistently higher in RT than in the fruit stored at LT, indicating that the color of the fruit in RT was brighter and changed to yellow more quickly. The trend in a* was the opposite, indicating that the fruits at LT were greener (**Figures 1D–F**). The TSS content of fruit stored at RT was significantly higher than that of fruit stored at LT, while the acid content of fruit stored at LT was higher than that of fruit stored at RT, and the ratio of TSS/acid was somewhat higher in fruit at RT, with the difference that after the transfer from LT to RT, it was higher than that of fruit stored at RT (**Figures 1G–I**).

### 3.2 Total volatile compounds of different temperature stored fruit

**Figure 2:**
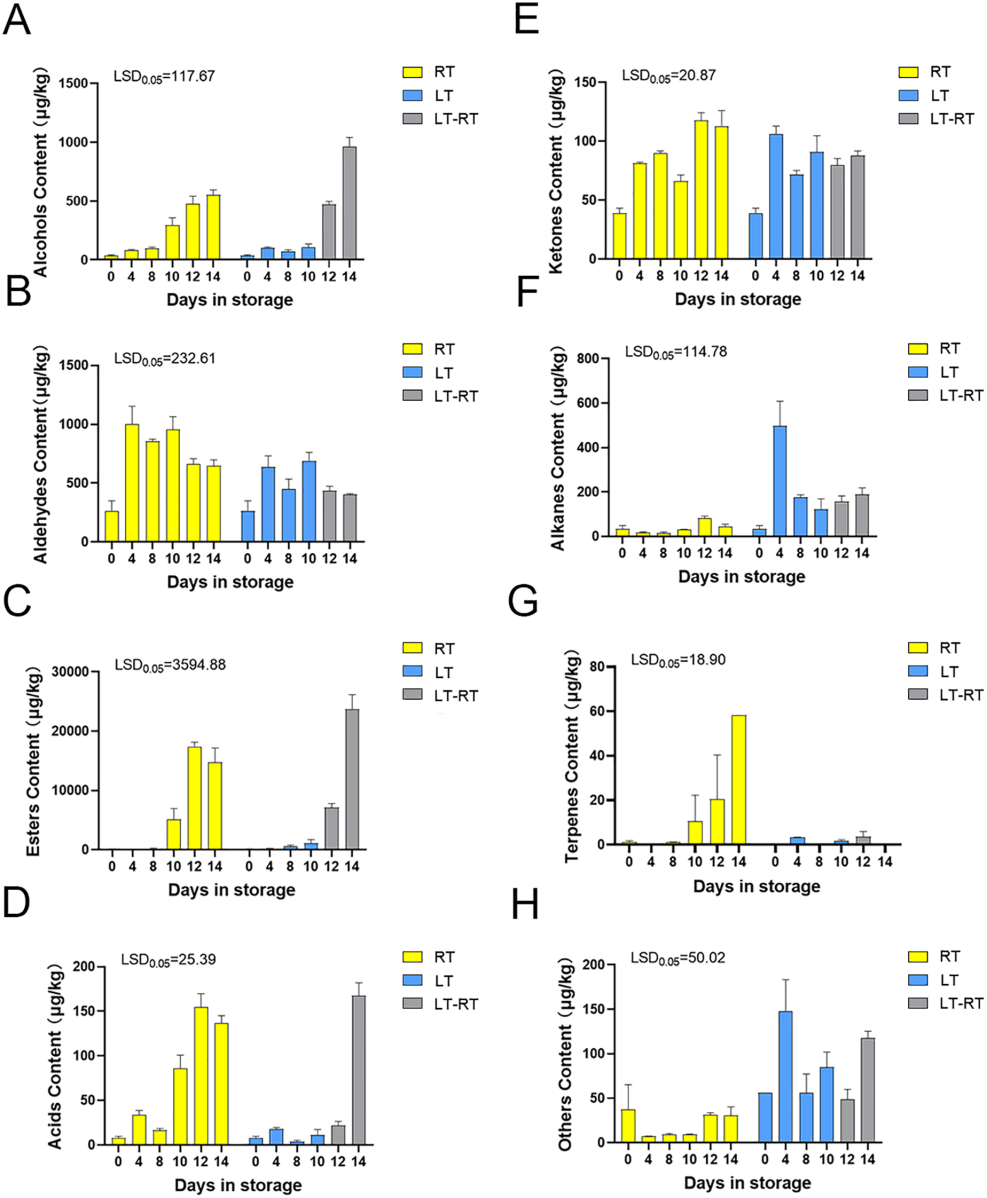
The total volatile compounds composition of ‘Docteur Jules Guyot’ pear stored at RT, LT, and LT-RT. **(A)** Content of Alcohols **(B)** Content of Aldehydes **(C)** Content of Esters **(D)** Content of Acids **(E)** Content of Ketones **(F)** Content of Alkanes **(G)** Content of Terpenes **(H)** Content of Others.

The detection and analysis of all volatile compounds in the fruit stored at RT, LT and LT-RT revealed that the main volatile compounds in the ‘Docteur Jules Guyot’ pear were alcohols, aldehydes, esters, acids, ketones, alkanes, terpenes and others (**Supplementary Table S1**). The total alcohol substance content of fruit stored at RT gradually increased with storage time, and the content of total alcoholic substances changed little at LT, being lower than that in fruit stored at RT, and then increased rapidly after transferring to RT, being higher than that in fruit stored at RT during the same time period (**Figure 2A**). Regarding the substances representative of the immature period of the fruit, the total aldehyde content gradually decreased with the ripening of the fruit, which showed the opposite trend to that of total alcohols (**Figure 2B**). The trends of total alcohol substances and total ester substances were consistent with those of ethylene production: the total ester content of the fruit in RT increased from 120.3 μg/kg on day 0 to 14,754.6 μg/kg on day 14, the total ester substance content did not change much in LT, and the content increased after transferring to RT, from 1,116.4 μg/kg to 23,705.2 μg/kg, which was more than the total ester content produced by fruit stored at RT, and the odor of the fruit was more intense as a result (**Figure 2C**). The acid content of the fruit at RT gradually increased, while the acid content of the fruit at LT remained lower than that at RT; however, after transfer to RT, the acid content increased rapidly (**Figure 2D**). There was no significant difference in the trend of ketones stored at RT and LT (**Figure 2E**). The change trend of alkanes in fruit stored at room temperature was not obvious, increasing for 4 days at low temperatures, then decreasing and remaining stable (**Figure 2F**). The content of terpenes in fruits stored at RT increased gradually, which improved the aroma of the fruit, with no significant change at low temperatures (**Figure 2G**). In addition to the main substances, such as alcohols, aldehydes, esters, acids, ketones, alkanes, and terpenes, very low amounts of other substances were present (**Figure 2H**). In the aroma of ‘Docteur Jules Guyot’ pear, esters are the most important substances, and the proportion of other substances in the total volatile compound content is much smaller than that of esters.

### 3.3 Representative volatile compounds in ‘Docteur Jules Guyot’ pear fruit

**Figure 3:**
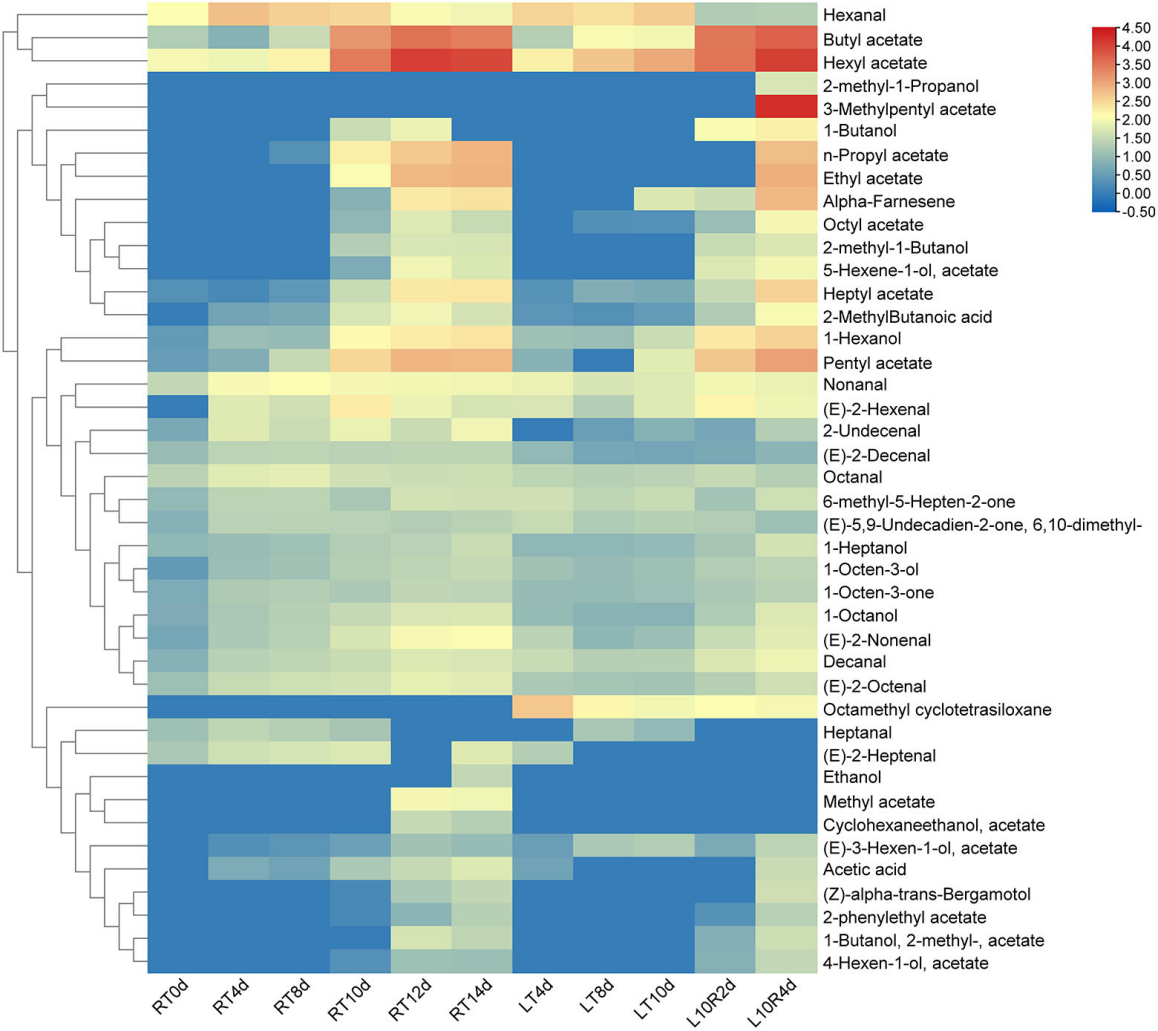
Heatmap of the corresponding volatiles screened during RT, LT, and LT-RT storage of pear fruit.

**Figure 4:**
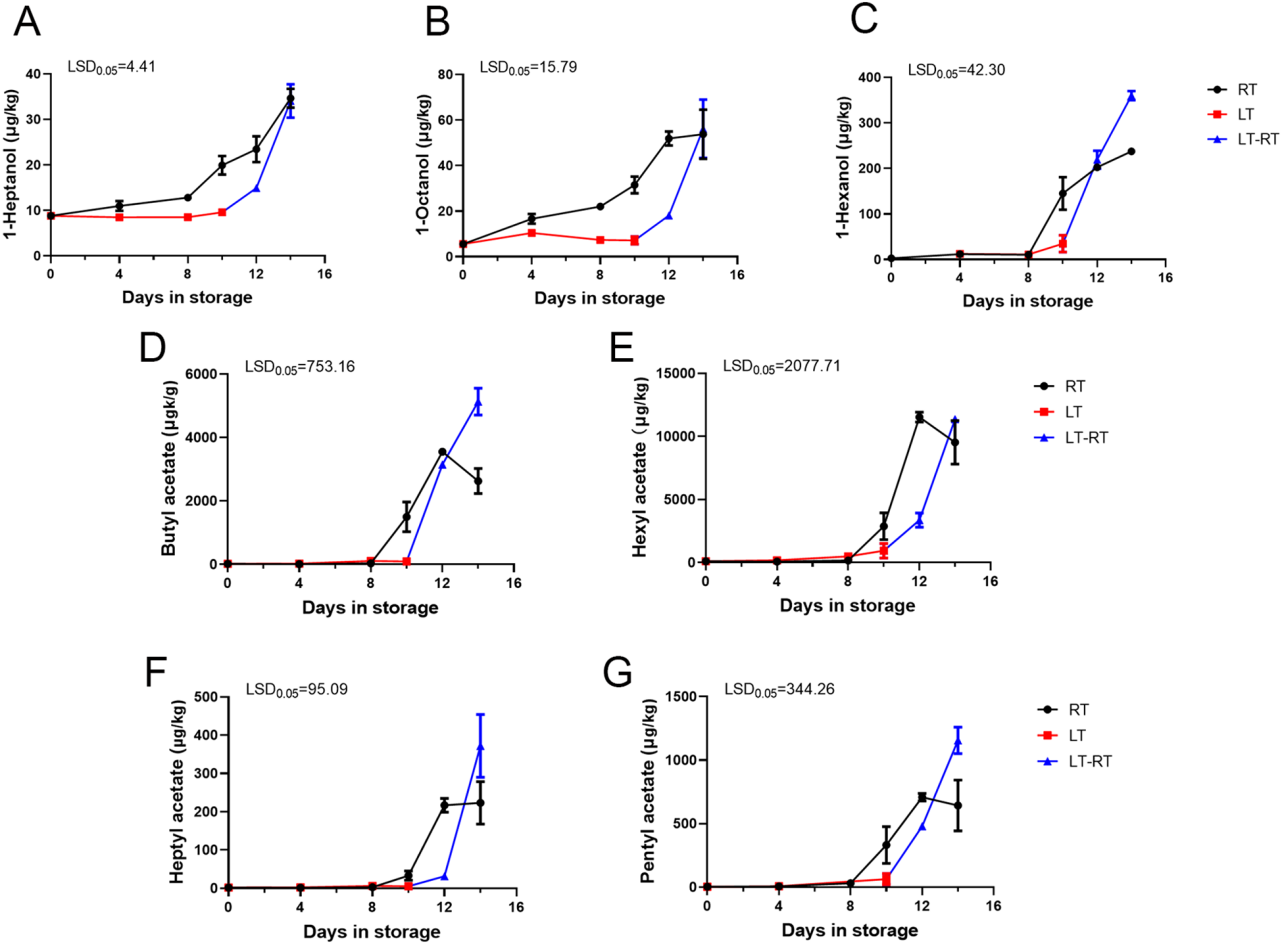
Characteristic volatile substance analysis of ‘Docteur Jules Guyot’ pear fruit. **(A–C)** Changes in the alcohol content at RT, LT, and LT-RT. **(D–G)** Changes in the ester content at RT, LT, and LT-RT. Error bars come from three repeated SE. The observed LSD values suggest the presence of LSD with a significance level of p = 0.05.

Through statistical analysis of all the volatile compounds detected in ‘Docteur Jules Guyot’ pear (**Supplementary Table S1**), according to the volatile compounds produced on different days, a substance content of more than 20 μg/kg was screened, and these compounds were analyzed using a heat map. To obtain the main characteristic aroma in ‘Docteur Jules Guyot’ pear, we continued to screen these data, under the condition that this substance must be produced at both RT and LT, and the content is more than 200 μg/kg (**Figure 3**). It can be seen that with the continuous ripening of fruit, the hexanal content gradually decreases, which reduces the grassy aroma of fruits during the immature stage. Among these, the substances with the greatest variation were esters. Butyl acetate, hexyl acetate, heptyl acetate, pentyl acetate, and alcohols increased significantly with fruit storage. Under RT storage, from day 0 to day 14, 1-heptanol increased from 8.8 μg/kg to 34.6 μg/kg (**Figure 4A**), 1-octanol from 5.6 μg/kg to 53.8 μg/kg (**Figure 4B**), 1-hexanol increased from 2.9 μg/kg to 237.6 μg/kg (**Figure 4C**), butyl acetate increased from 19.6 μg/kg to 2,627.9 μg/kg (**Figure 4D**), hexyl acetate increased from 102.7 μg/kg to 9,526.2 μg/kg (**Figure 4E**), heptyl acetate increased from 2.0 μg/kg to 223.3 μg/kg (**Figure 4F**), and pentyl acetate increased from 3.2 μg/kg to 642.6 μg/kg (**Figure 4G**). The contents of different substances in fruit stored at RT began to increase from day 8, which was significantly higher than that of fruit stored at LT. In the case of fruit stored at LT, the content remained at a low level until the 10 days, and increased rapidly after transfer to RT (**Figure 4**). It was shown that the production of aroma was inhibited by LT, and after transfer from LT to RT, the same number of esters was released as in samples stored at RT.

### 3.4 Expression of genes involved in ester synthesis

**Figure 5:**
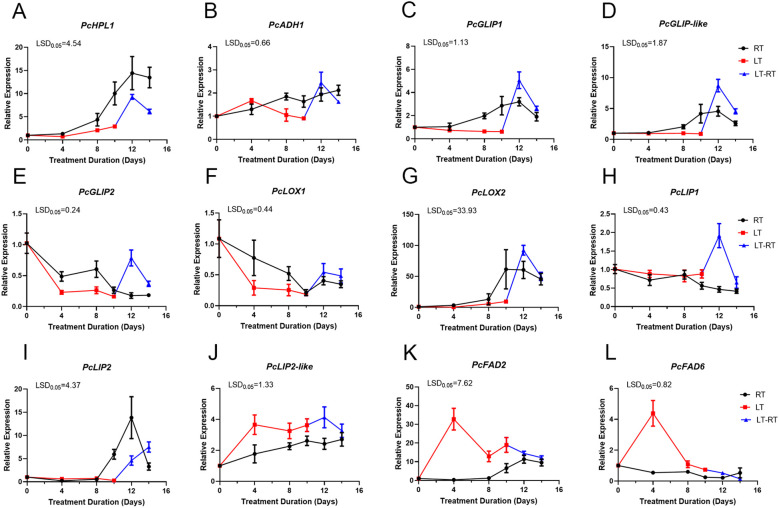
Expression of **(A)** *PcHPL1* **(B)** *PcADH1* **(C)** *PcGLIP1* **(D)** *PcGLIP-like* **(E)** *PcGLIP2* **(F)** *PcLOX1* **(G)** *PcLOX2* **(H)** *PcLIP1* **(I)** *PcLIP2* **(J)** *PcLIP2-like* **(K)** *PcFAD2* and **(L)** *PcFAD6* associated with ester synthesis at RT, LT, and LT-RT. Error bars come from three repeated SE. The observed LSD values suggest the presence of LSD with a significance level of p = 0.05.

In order to verify the key genes associated with ester production in ‘Docteur Jules Guyot’ pear, based on the preliminary transcriptome data analysis of our research group (Dai et al., 2023), we screened 12 candidate ester biosynthesis pathway genes with significant differential expression during postharvest softening (log2 fold change (FC)| >1, FPKM >20). The expression levels of hydroperoxide lyase (HPL), alcohol dehydrogenase (ADH), GDSL lipase (GLIP), lipase (LIP), lipoxygenase (LOX), and fatty acid desaturase (FAD) genes in ‘Docteur Jules Guyot’ pear were examined by qRT-PCR (**Figure 5**). The expression levels of *PcHPL1*, *PcADH1*, *PcGLIP1*, *PcGLIP-like*, and *PcLOX2* in RT-stored pear fruit gradually increased after 4 days of storage; on day 12 at RT, the expression levels of these genes peaked after 2 days of conversion from LT to RT. Overall, the expression at RT was significantly higher than that at LT, but after 2 days of LT-RT, it was higher than that at RT (**Figures 5A–D, G**). The expression levels of *PcGLIP2* and *PcLOX1* were gradually decreased in fruit, and the expression levels at RT were higher than LT, after the transition from LT to RT, the expression of *PcGLIP2* and *PcLOX1* increased rapidly and was higher than that in RT (**Figures 5E, F**). The expression of *PcLIP1* showed little change before 8 days of storage at RT and began to decline gradually after 8 days. Expression changed little after 10 days of storage at LT, increased rapidly after transfer to RT, and then declined (**Figure 5H**). The expression of *PcLIP2* showed little change before 8 days of storage at RT and then increased gradually after 8 days. The expression of *PcLIP1* and *PcLIP2* peaked on day 12, showed little change when stored in LT for 10 days, and then increased rapidly after transfer to RT (**Figures 5H, I**). The expression of *PcLIP2-like*, *PcFAD2*, and *PcFAD6* genes was induced by LT (**Figures 5J–L**).

### 3.5 Relationships between the main characteristic aroma substances and related genes in ‘Docteur Jules Guyot’ pear

**Figure 6:**
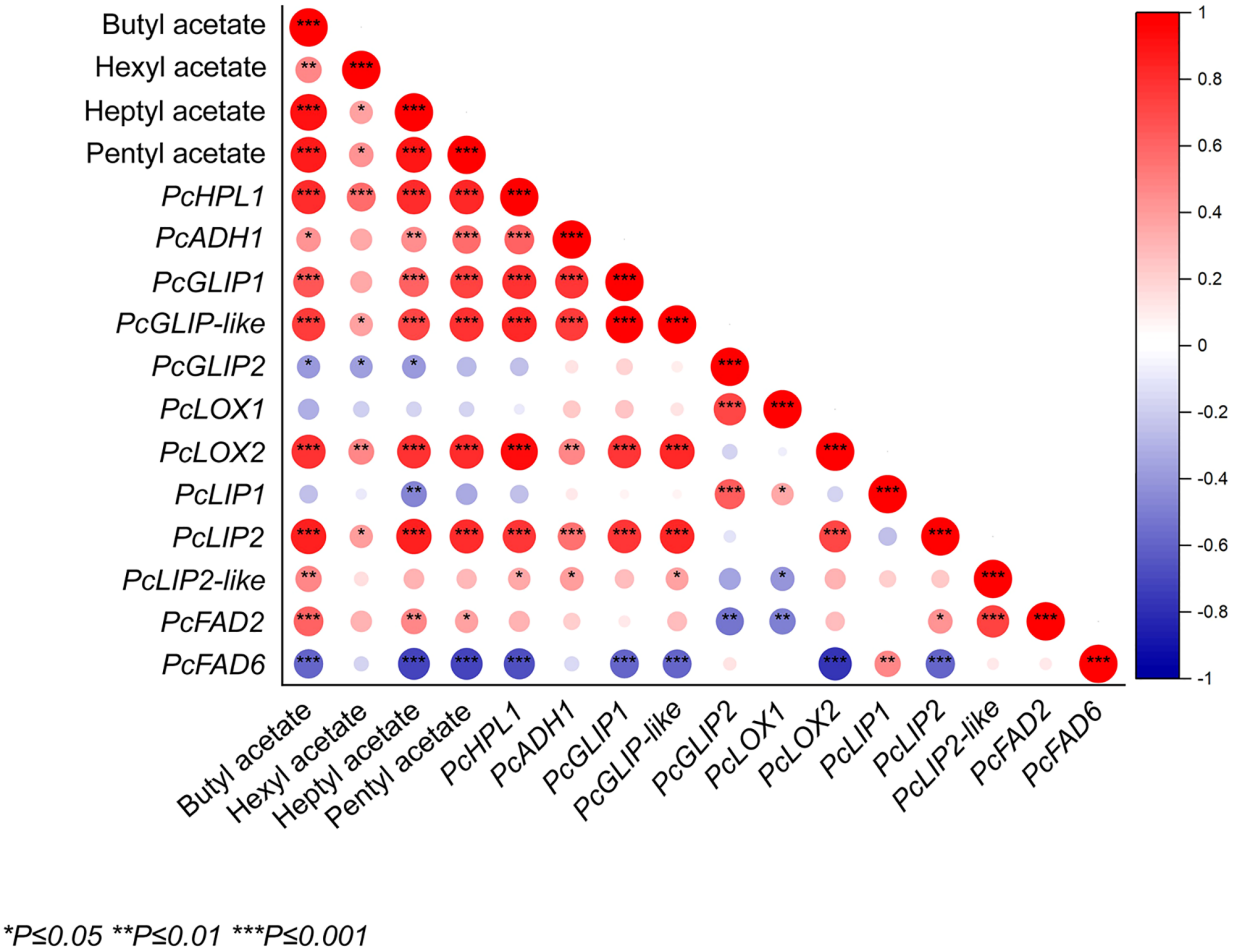
The correlation between esters and expression levels of ester synthesis genes in ‘Docteur Jules Guyot’ pear fruit. Positive and negative correlations are shown in red and blue, respectively (*p <0.05, **p <0.01, ***p <0.001).

The main characteristic substances of fruit aroma production were correlated with the expression of related genes. The results showed that butyl acetate, heptyl acetate, and pentyl acetate were positively correlated with *PcHPL1*, *PcGLIP1*, *PcGLIP-like*, *PcLOX2*, and *PcLIP2* (**Figure 6**). Butyl acetate, heptyl acetate, and pentyl acetate positively correlated with *PcHPL1*, *PcLOX2*, and *PcLIP2*, with correlation coefficients greater than 0.7 (**Figure 6**). The gene with the highest negative correlation with butyl acetate, heptyl acetate, and pentyl acetate was *PcFAD6*, with correlation coefficients of −0.59, −0.71, and −0.71, respectively. The gene with the highest positive correlation with hexyl acetate was *PcHPL1* with a correlation coefficient of 0.57 and the gene with the highest negative correlation was *PcGLIP2* with a correlation coefficient of −0.37 (**Figure 6**). Butyl acetate was significantly positively correlated with *PcFAD2* and pentyl acetate was positively correlated with *PcADH1*. Similarly, all of these substances were significantly negatively correlated with *PcFAD6*. Hexyl acetate was significantly positively associated with *PcHPL1*.

## 4 Discussion

The ‘Docteur Jules Guyot’ pear is one of the most representative varieties of European pears. A notable feature of European pears is that, after harvest, they undergo a post-ripening process, during which the fruits gradually soften and release a rich aroma. However, during the long process of species evolution, there are still certain differences among different European pear varieties. For example, existing studies have shown that different European pear varieties can be classified into fruity and aliphatic aroma types (Chen et al., 2018). However, regardless of the specific category to which they belong, all European pear varieties share one common point: their volatile compounds are mainly concentrated in chemicals such as esters, alcohols, alkanes, acids, ketones, terpenes, and aldehydes. This is highly consistent with our research results, further verifying the reliability of the experimental data. Although the research conclusion of the ‘Docteur Jules Guyot’ pear cannot be simply applied directly to other varieties, however, through in-depth analysis of the ‘Docteur Jules Guyot’ pear, we can better understand the aroma formation mechanism of European pears as a whole and the scientific principles behind it, thus laying a solid foundation for future research on other varieties.

Physiological and internal changes in European pears during postharvest ripening and softening are accompanied by a decrease in firmness, color differences, and changes in sugar and acid content. The aroma is also an important characteristic of fruit ripening, providing greater perceived value for the fruit itself and promoting consumption (Song and Forney, 2008). Banana is a typical respiratory fruit that undergoes changes in respiration, color, texture, and volatile production during ripening (Yang et al., 2011). In the green stage of banana ripening, the total volatiles were mainly aldehydes, with significant amounts of esters detected in the ripening stage, and the ethanol content increased at the over-ripening stage (Zhu et al., 2010). Fruit preservation has always been an important aspect to affect fruit aroma, and the most commonly used fruit preservation method is low-temperature storage (Brosnan and Sun, 2001; Zhang et al., 2017). With the development of aroma research, it was found that the content of total volatile substances decreased during low-temperature storage (Infante et al., 2008). In the study of ‘Nanguo’ pear aroma content at low temperatures, the longer the storage time, the lower the volatile content, and the more pronounced the tendency to fade. This phenomenon also occurs in other fruits, including tomatoes, peaches, citrus, and mangoes (de León-Sánchez et al., 2009; Zhang et al., 2011; Günther et al., 2015). These results indicate that low temperatures affect the production of fruit aromas. The volatile content of ‘Docteur Jules Guyot’ pear fruit at low temperatures was also significantly lower than that at room temperature (**Figure 2**). Furthermore, during postharvest storage of fruits, environmental factors can affect the generation of volatile substances. In grapes, insufficient light can lead to a significant reduction in the content and type of volatile compounds (Ma et al., 2021). In our study, an environmental control system was used to ensure that temperature was the only variable, thereby improving the reliability of the results.

In ‘Nanguo’ pear, ethyl caproate accounted for the largest proportion of aroma components, while ethyl acetate, ethyl butyrate and hexyl acetate were identified as the main volatile components in the fruit (Zhou et al., 2015). The high content of aldehydes highlights the apple-like aroma, and ester substances highlight the fruit aroma, such as some alcohol esters, including ethyl acetate, which contribute flavor compounds to the fruit aroma (Gan et al., 2014). From the heatmap, it can be seen that the greatest change in substance content in ‘Docteur Jules Guyot’ pear was also ester (**Figure 3**). In ‘Nanguo’ pear, ester was also a key volatile compound that determine the typical aroma of the fruit (Li et al., 2022). When stored at room temperature, the changes in the content of butyl acetate, hexyl acetate, heptyl acetate, and amyl acetate were the most significant (**Figure 4**). These ester compounds are the main components that form the fruit aroma, and their concentrations directly affect the flavor of the fruit. Meanwhile, the contents of these substances showed a reverse exceeding phenomenon after 10 days at low temperatures for 4 days at room temperature. This means that compared with 14 days of storage at room temperature, the treatment of low temperature followed by room temperature can prompt the fruits to produce more aroma substances. However, this treatment method did not have a significant effect on the hardness and sugar–acid ratio of the fruits (**Figure 1**). By adopting the storage strategy of alternating “low temperature–room temperature,” merchants can effectively enhance the aroma level of the fruits without sacrificing fruit quality, thereby strengthening the market competitiveness of the products.

Fatty acids are the precursors for the formation of most fruit aromas, and the content of the derived volatile compounds, such as alcohols, aldehydes, esters, acids, and ketones, affects the fruit flavor (El Hadi et al., 2013). In ‘Nanguo’ pear, high LOX activity and expression of *PuLOX1* in fruit stored at low temperatures promoted the production of ethyl hexanoate and hexyl acetate substances (León et al., 2002; Zhang and Tian, 2010). Studies have shown that the production of ethyl acetate and hexyl acetate occurs via the fatty acid metabolic pathway (Schwab et al., 2008). In ‘Docteur Jules Guyot’ pear, *PcLOX1* was downregulated and *PcLOX2* was upregulated during postharvest ripening (**Figure 5**). Under the action of ADH, aldehydes can be converted into hexenol and hexanol, and the level of ADH expression may be correlated with the amount of aroma (Buttery et al., 1971). In our study, we found that *PcADH1* was upregulated during RT storage (**Figure 5**). Linoleic acid is an important precursor for the biosynthesis of flavor volatiles and is catalyzed by fatty acid desaturase (FAD) (Dehghan Nayeri and Yarizade, 2014; Jin et al., 2022). Studies on peaches revealed that the production of flavor volatiles such as hexenal and hexenol within the fruit was promoted by the catalysis of *PpFAD3-1* (Jin et al., 2022). By overexpressing *FAD3* and *FAD7* in transgenic tomato plants, the tolerance of transgenic tomato plants to cold storage treatment was improved (Domínguez et al., 2010). Similarly in our experimental results, it can be seen that the expression of *PcFAD2* and *PcFAD6* were much higher under low temperature storage than in room temperature (**Figure 5**).

In the results of principal component analysis and correlation analysis of ‘Nanguo’ pear, *PuLOX3* and *PuAAT* were positively correlated with ester formation (Luo et al., 2021). Analysis of volatile substances in ‘Docteur Jules Guyot’ pear with related genes revealed that the *PcHPL1*, *PcLOX2*, and *PcLIP2* genes were highly correlated with the synthesis of ester substances (**Figure 6**). Similarly, the LOX pathway is involved in ester biosynthesis in melting peaches (Zhang et al., 2011). The transcript levels of *PuFAD2.1*, *PuLOX2-1*, *PuLOX5*, *PuADH1*, and *PuAAT* in ‘Nanguo’ pear were significantly positive correlated with the contents of oleic, linoleic and linolenic acids in fruit at 10 DAH, and the trends of their contents were consistent with esters (Li et al., 2022).

## 5 Conclusion

By analyzing the volatiles of ‘Docteur Jules Guyot’ pear stored at low temperature, room temperature and low temperature to room temperature at different stages of ripening using solid-phase microextraction (SPME) with gas chromatography–mass spectrometry (GC–MS). The identified volatile substances were classified as alcohols, aldehydes, esters, acids, ketones, alkanes, and terpenes. The ester, alcohol, and acid contents gradually increased with storage time at room temperature. Under low-temperature conditions, the content of these three types of volatile substances either increased or decreased slightly. However, after returning to room temperature, their contents rapidly rebound over a short period. Butyl hexanoate, hexyl acetate, heptyl acetate, and amyl acetate may be the main esters affecting the aroma characteristics of ‘Docteur Jules Guyot’ pear. Correlation analysis showed that the expression levels of *PcHPL1*, *PcADH1*, *PcGLIP1*, *PcGLIP-like*, *PcLOX2*, *PcLIP2*, and *PcFAD2* were positively correlated with the content of major esters. Therefore, we speculated that these genes may play key roles in the synthesis of esters in ‘Docteur Jules Guyot’ pear.

## Data Availability

The original contributions presented in the study are included in the article/**Supplementary Material**. Further inquiries can be directed to the corresponding author.

## Supplementary material

Supplementary Table 2 Overview of the GC–MS results.

## References

[B1] Ayala-Zavala J. F. Wang S. Y. Wang C. Y. González-Aguilar G. A. (2004). Effect of storage temperatures on antioxidant capacity and aroma compounds in strawberry fruit. LWT-Food Sci. Technol. 37, 687–695. doi: 10.1016/j.lwt.2004.03.002

[B2] Brosnan T. Sun D. W. (2001). Precooling techniques and applications for horticultural products-a review. Int. J. Refrig 24, 154–170. doi: 10.1016/s0140-7007(00)00017-7

[B3] Buttery R. G. Seifert R. M. Guadagni D. G. Ling L. C. (1971). Characterization of additional volatile components of tomato. J. Agr. Food Chem. 19, 524–529. doi: 10.1021/jf60175a011

[B4] Chen Y. Y. Yin H. Wu X. Shi X. J. Qi K. J. Zhang S. L. (2018). Comparative analysis of the volatile organic compounds in mature fruits of 12 Occidental pear (*Pyrus communis* L.) cultivars. Sci. Hortic. 240, 239–248. doi: 10.1016/j.scienta.2018.06.014

[B5] Contreras C. Beaudry R. (2013). Lipoxygenase-associated apple volatiles and their relationship with aroma perception during ripening. Postharvest Biol. Tec 82, 28–38. doi: 10.1016/j.postharvbio.2013.02.006

[B6] Dai X. N. Jiang F. D. Li Q. Y. Yu X. H. Xu X. F. Cao W. L.. (2023). Transcriptome analysis for key softening-related genes in ‘Docteur Jules Guyot’ pear (*Pyrus communis* L.). Postharvest Biol. Tec 205, 112484. doi: 10.1016/j.postharvbio.2023.112484

[B7] Dehghan Nayeri F. Yarizade K. (2014). Bioinformatics study of delta-12 fatty acid desaturase 2 (FAD2) gene in oilseeds. Mol. Biol. Rep. 41, 5077–5087. doi: 10.1007/s11033-014-3373-5 24816719

[B8] de León-Sánchez F. D. Pelayo-Zaldívar C. Rivera-Cabrera F. Ponce-Valadez M. Ávila-Alejandre X. Fernández F. J.. (2009). Effect of refrigerated storage on aroma and alcohol dehydrogenase activity in tomato fruit. Postharvest Biol. Tec 54, 93–100. doi: 10.1016/j.postharvbio.2009.07.003

[B9] Dixon J. Hewett E. W. (2000). Factors affecting apple aroma/flavour volatile concentration: a review, New Zeal J Crop Hort. N. Z. J. Crop Hortic. Sci. 28, 155–173. doi: 10.1080/01140671.2000.9514136

[B10] Domínguez T. Hernández M. L. Pennycooke J. C. Jiménez P. Martínez-Rivas J. M. Sanz C.. (2010). Increasing ω-3 desaturase expression in tomato results in altered aroma profile and enhanced resistance to cold stress. Plant Physiol. 153, 655–665. doi: 10.1104/pp.110.154815 20382895 PMC2879794

[B11] El Hadi M. A. M. Zhang F. J. Wu F. F. Zhou C. H. Tao J. (2013). Advances in fruit aroma volatile research. Molecules 18, 8200–8229. doi: 10.3390/molecules18078200 23852166 PMC6270112

[B12] Gan H. H. Soukoulis C. Fisk I. (2014). Atmospheric pressure chemical ionisation mass spectrometry analysis linked with chemometrics for food classification–A case study: Geographical provenance and cultivar classification of monovarietal clarified apple juices. Food Chem. 146, 149–156. doi: 10.1016/j.foodchem.2013.09.024 24176326

[B13] Günther C. S. Marsh K. B. Winz R. A. Harker R. F. Wohlers M. W. White A.. (2015). The impact of cold storage and ethylene on volatile ester production and aroma perception in ‘Hort16A’ kiwifruit. Food Chem. 169, 5–12. doi: 10.1016/j.foodchem.2014.07.070 25236191

[B14] Infante R. Farcuh M. Meneses C. (2008). Monitoring the sensorial quality and aroma throμgh an electronic nose in peaches during cold storage. J. Sci. Food Agric. 88, 2073–2078. doi: 10.1002/jsfa.3316

[B15] Jetti R. R. Yang E. Kurnianta A. Finn C. Qian M. C. (2007). Quantification of selected aroma-active compounds in strawberries by headspace solid-phase microextraction gas chromatography and correlation with sensory descriptive analysis. J. Food science. 72, S487–S496. doi: 10.1111/j.1750-3841.2007.00445.x 17995662

[B16] Jin Z. N. Wang J. J. Cao X. M. Wei C. Y. Kuang J. F. Chen K. S.. (2022). Peach fruit PpNAC1 activates PpFAD3-1 transcription to provide ω-3 fatty acids for the synthesis of short-chain flavor volatiles. Hortic 9, uhac085. doi: 10.1093/hr/uhac085 PMC9172071 35685221

[B17] Karlova R. Chapman N. David K. Angenent G. C. Seymour G. B. De Maagd R. A. (2014). Transcriptional control of fleshy fruit development and ripening. J. Exp. Bot. 65, 4527–4541. doi: 10.1093/jxb/eru316 25080453

[B18] Lee J. H. J. Jayaprakasha G. K. Rush C. M. Crosby K. M. Patil B. S. (2018). Production system influences volatile biomarkers in tomato. Metabolomics 14, 1–13. doi: 10.1007/s11306-018-1385-1 30830380

[B19] León J. Royo J. Vancanneyt G. Sanz C. Silkowski H. Griffiths G.. (2002). Lipoxygenase H1 gene silencing reveals a specific role in supplying fatty acid hydroperoxides for aliphatic aldehyde production. J. Biol. Chem. 277, 416–423. doi: 10.1074/jbc.m107763200 11675388

[B20] Li J. Z. Dai X. N. Li Q. Y. Jiang F. D. Xu X. F. Guo T. T.. (2023). Low temperatures inhibit the pectin degradation of ‘Docteur Jules Guyot’pear (*Pyrus communis* L.). Int. J. Biol. Macromol 242, 124719. doi: 10.1016/j.ijbiomac.2023.124719 37150373

[B21] Li X. J. Qi L. Y. Zang N. N. Zhao L. H. Sun Y. Q. Huang X. T.. (2022). Integrated metabolome and transcriptome analysis of the regulatory network of volatile ester formation during fruit ripening in pear. Plant Physiol. Bioch. 185, 80–90. doi: 10.1016/j.plaphy.2022.04.030 35661588

[B22] Liu Z. C. Liang T. Kang C. Y. (2023). Molecular bases of strawberry fruit quality traits: Advances, challenges, and opportunities. Plant Physiol. 193, 900–914. doi: 10.1093/plphys/kiad376 37399254

[B23] Lu P. F. Huang L. Q. Wang C. Z. (2012). Identification and field evaluation of pear fruit volatiles attractive to the oriental fruit moth, Cydia molesta. J. Chem. Ecol. 38, 1003–1016. doi: 10.1007/s10886-012-0152-4 22730107

[B24] Luo M. L. Zhou X. Sun H. J. Zhou Q. Ge W. Y. Sun Y. Y.. (2021). Insights into profiling of volatile ester and LOX-pathway related gene families accompanying post-harvest ripening of ‘Nanguo’ pears. Food Chem. 335, 127665. doi: 10.1016/j.foodchem.2020.127665 32738530

[B25] Ma Z. Yang S. Mao J. (2021). Effects of shading on the synthesis of volatile organic compounds in ‘Marselan’ Grape berries (Vitis vinifera L.) J. Plant Growth Regul. 40, 679–693. doi: 10.1007/s00344-020-10123-2

[B26] Schwab W. Davidovich-Rikanati R. Lewinsohn E. (2008). Biosynthesis of plant-derived flavor compounds. Plant J. 54, 712–732. doi: 10.1111/j.1365-313X.2008.03446.x 18476874

[B27] Song X. Y. Dai F. Yao J. R. Li Z. Huang Z. P. Liu H. J.. (2023). Characterization of the volatile profile of feijoa (Acca sellowiana) fruit at different ripening stages by HS-SPME-GC/MS. LWT-Food Sci. Technol. 184, 115011. doi: 10.1016/j.lwt.2023.115011

[B28] Song J. Forney C. F. (2008). Flavour volatile production and regulation in fruit, Can. J. Plant Sci. 88, 537–550. doi: 10.4141/cjps07170

[B29] Tobaruela E. D. C. Gomes B. L. Bonato V. C. D. B. Lima E. S. D. Freschi L. Purgatto E. (2021). Ethylene and auxin: hormonal regulation of volatile compound production during tomato (*Solanum lycopersicum* L.) fruit ripening. Front. Plant Sci. 12. doi: 10.3389/fpls.2021.765897 PMC8702562 34956263

[B30] Villatoro C. Altisent R. Echeverría G. Graell J. López M. L. Lara I. (2007). Changes in biosynthesis of aroma volatile compounds during on-tree maturation of ‘Pink Lady^®^’ apples, Postharvest Biol Tec 47, 286–295. doi: 10.1016/j.postharvbio.2007.07.003

[B31] Wang S. S. Saito T. Ohkawa K. Ohara H. Suktawee S. Ikeura H.. (2018). Abscisic acid is involved in aromatic ester biosynthesis related with ethylene in green apples. J. Plant Physiol. 221, 85–93. doi: 10.1016/j.jplph.2017.12.007 29268086

[B32] Watharkar R. B. Pu Y. F. Ismail B. B. Srivastava B. Srivastav P. P. Liu D. H. (2020). Change in physicochemical characteristics and volatile compounds during different stage of banana (Musa nana Lour vs. Dwarf Cavendish) ripening. J. Food Meas. Charact 14, 2040–2050. doi: 10.1007/s11694-020-00450-z

[B33] Xi W. P. Zhang B. Shen J. Y. Xu C. J. Chen K. S. (2012). Intermittent warming alleviated the loss of peach fruit aroma-related esters by regulation of AAT during cold storage. Postharvest Biol. Tec 74, 42–48. doi: 10.1016/j.postharvbio.2012.07.003

[B34] Yang X. T. Song J. Fillmore S. Pang X. Q. Zhang Z. Q. (2011). Effect of high temperature on color, chlorophyll fluorescence and volatile biosynthesis in green-ripe banana fruit. Postharvest Biol. Tec. 62, 246–257. doi: 10.1016/j.postharvbio.2011.06.011

[B35] Zhang C. F. Tian S. P. (2010). Peach fruit acquired tolerance to low temperature stress by accumulation of linolenic acid and N-acylphosphatidylethanolamine in plasma membrane. Food Chem. 120, 864–872. doi: 10.1016/j.foodchem.2009.11.029

[B36] Zhang B. Xi W. P. Wei W. W. Shen J. Y. Ferguson I. Chen K. S. (2011). Changes in aroma-related volatiles and gene expression during low temperature storage and subsequent shelf-life of peach fruit. Postharvest Biol. Tec. 60, 7–16. doi: 10.1016/j.postharvbio.2010.09.012

[B37] Zhang A. D. Zhang Q. Y. Li J. Z. Gong H. S. Fan X. G. Yang Y. Q.. (2020). Transcriptome co-expression network analysis identifies key genes and regulators of ripening kiwifruit ester biosynthesis. BMC Plant Biol. 20, 1–12. doi: 10.1186/s12870-020-2314-9 32138665 PMC7059668

[B38] Zhang Z. K. Zhu Q. G. Hu M. J. Gao Z. Y. An F. Li M.. (2017). Low-temperature conditioning induces chilling tolerance in stored mango fruit. Food Chem. 219, 76–84. doi: 10.1016/j.foodchem.2016.09.123 27765262

[B39] Zhao J. T. Sauvage C. Zhao J. H. Bitton F. Bauchet G. Liu D.. (2019). Meta-analysis of genome-wide association studies provides insights into genetic control of tomato flavour. Nat. Commun. 10, 1534. doi: 10.1038/s41467-019-09462-w 30948717 PMC6449550

[B40] Zhou X. Dong L. Li R. Zhou Q. Wang J. W. Ji S. J. (2015). Low temperature conditioning prevents loss of aroma-related esters from ‘Nanguo’ pears during ripening at room temperature. Postharvest Biol. Tec. 100, 23–32. doi: 10.1016/j.postharvbio.2014.09.012

[B41] Zhu H. Li X. P. Yuan R. C. Chen Y. F. Chen W. X. (2010). Changes in volatile compounds and associated relationships with other ripening events in banana fruit. J. Hortic. Sci. Biotech. 85, 283–288. doi: 10.1080/14620316.2010.11512669

